# Complications of Autologous Stem Cell Transplantation in Multiple Myeloma: Results from the CALM Study

**DOI:** 10.3390/jcm11123541

**Published:** 2022-06-20

**Authors:** Anna Waszczuk-Gajda, Olaf Penack, Giulia Sbianchi, Linda Koster, Didier Blaise, Péter Reményi, Nigel Russell, Per Ljungman, Marek Trneny, Jiri Mayer, Simona Iacobelli, Guido Kobbe, Christof Scheid, Jane Apperley, Cyrille Touzeau, Stig Lenhoff, Esa Jantunen, Achilles Anagnostopoulos, Laura Paris, Paul Browne, Catherine Thieblemont, Nicolaas Schaap, Jorge Sierra, Ibrahim Yakoub-Agha, Laurent Garderet, Jan Styczynski, Helene Schoemans, Ivan Moiseev, Rafael F. Duarte, Zinaida Peric, Silvia Montoto, Anja van Biezen, Malgorzata Mikulska, Mahmoud Aljurf, Tapani Ruutu, Nicolaus Kröger, Curly Morris, Christian Koenecke, Stefan Schoenland, Grzegorz W. Basak

**Affiliations:** 1 Department of Hematology, Transplantation and Internal Medicine, University Clinical Centre—The Medical University of Warsaw, 02-097 Warsaw, Poland; grzegorz.basak@wum.edu.pl; 2 Charité Universitätsmedizin Berlin, 10771 Berlin, Germany; olaf.penack@charite.de; 3 Tor Vergata University, 00133 Rome, Italy; giulia.sbianchi@uniroma2.it (G.S.); simona.iacobelli@ebmt.org (S.I.); 4 EBMT Data Office Leiden, 2333 AA Leiden, The Netherlands; l.koster@lumc.nl (L.K.); clwpebmt@lumc.nl (A.v.B.); 5 Institut Paoli Calmettes, 13009 Marseille, France; blaised@ipc.unicancer.fr; 6 Dél-Pesti Centrumkórház, 1097 Budapest, Hungary; premenyi@dpckorhaz.hu; 7 Nottingham University, Nottingham NG7 2QL, UK; nigel.russell@nottingham.ac.uk; 8 Department of Cellular Therapy and Allogeneic Stem Cell Transplantation, Karolinska Comprehensive Cancer Center, Karolinska University Hospital Huddinge, 17177 Stockholm, Sweden; per.ljungman@ki.se; 9 Division of Hematology, Department of Medicine Huddinge, Karolinska Institute, 171 77 Stockholm, Sweden; 10 University Hospital, 12808 Prague, Czech Republic; trneny@cesnet.cz; 11 University Hospital Brno, 62500 Brno, Czech Republic; mayer.jiri@fnbrno.cz; 12 Heinrich Heine Universitaet, 40225 Duesseldorf, Germany; kobbe@med.uni-duesseldorf.de; 13 University of Cologne, 50923 Cologne, Germany; c.scheid@uni-koeln.de; 14 Imperial College, London SW7-2BX, UK; j.apperley@imperial.ac.uk; 15 CHU Nantes, 44000 Nantes, France; cyrille.touzeau@chu-nantes.fr; 16 Skanes University Hospital, 23262 Lund, Sweden; stig.lenhoff@skane.se; 17 Department of Medicine, University of Eastern Finland and Hospital District of North Carelia, Kuopio University Hospital, 70211 Kuopio, Finland; esa.jantunen@kuh.fi; 18 George Papanicolaou General Hospital, 57010 Thessaloniki, Greece; achanagh@gmail.com; 19 Division of Hematology, SST Papa Giovanni XXIII, 24127 Bergamo, Italy; lparis@asst-pg23.it; 20 Hope Directorate, D08 NHY1 Dublin, Ireland; pbrowne@stjames.ie; 21 Hôpital St. Louis, 75010 Paris, France; catherine.thieblemont@aphp.fr; 22 Radboud University Medical Centre, Department of Hematology, 6525 GA Nijmegen, The Netherlands; michel.schaap@radboudumc.nl; 23 Hospital Santa Creu i Sant Pau, 08001 Barcelona, Spain; jsierra@santpau.cat; 24 CHU de Lille, 59000 Lille, France; ibrahim.yakoubagha@chru-lille.fr; 25 Centre de Recherche Saint-Antoine, Sorbonne Université-INSERM, UMR_S 938, 75013 Paris, France; laurent.garderet@aphp.fr; 26 Département d’Hématologie et de Thérapie Cellulaire, Assistance Publique-Hôpitaux de Paris, Hôpital Pitié Salpetrière, 75012 Paris, France; 27 Department of Pediatric Hematology and Oncology, Collegium Medicum UMK, 85-067 Bydgoszcz, Poland; jstyczynski@cm.umk.pl; 28 Department of Hematology, University Hospitals Leuven, 3000 Leuven, Belgium; helene.schoemans@uzleuven.be; 29 Department of Public Health and Primary Care, ACCENT VV, KU Leuven—University of Leuven, 3000 Leuven, Belgium; 30 R.M. Gorbacheva Memorial Institute of Hematology, Oncology and Transplantation, Pavlov University, 197022 Saint-Petersburg, Russia; moisiv@mail.ru; 31 Hospital Universitario Puerta de Hierro Majadahonda—Universidad Autónoma de Madrid, 28222 Madrid, Spain; rduarte.work@gmail.com; 32 Department of Internal Medicine, School of Medicine, University of Zagreb, 10000 Zagreb, Croatia; zinaida.peric@mef.hr; 33 Department of Haemato-Oncology, St Bartholomew’s Hospital, Barts Health NHS Trust, London EC1A 7BE, UK; s.montoto@qmul.ac.uk; 34 Division of Infectious Diseases, Department of Health Sciences (DISSAL), University of Genoa, 16121 Genoa, Italy; m.mikulska@unige.it; 35 Division of Infectious Diseases, IRC CS Ospedale Policlinico San Martino, 16132 Genoa, Italy; 36 Section of Adult Haematolgy/BMT, King Faisal Specialist Hospital & Research Centre Oncology, Riyadh 11564, Saudi Arabia; maljurf@kfshrc.edu.sa; 37 Comprehensive Cancer Center, Department of Hematology, Helsinki University Hospital and University of Helsinki, 00290 Helsinki, Finland; tapani.ruutu@hus.fi; 38 Clinical Research Institute, Helsinki University Hospital and University of Helsinki, 00280 Helsinki, Finland; 39 University Hospital Eppendorf, 20246 Hamburg, Germany; nkroeger@uke.de; 40 Belfast City Hospital, Belfast BT9 7AB, UK; curlymorris_cliff@yahoo.com; 41 Department of Hematology, Hemostasis, Oncology and Stem Cell Transplantation, Hannover Medical School, 30625 Hannover, Germany; koenecke.christian@mh-hannover.de; 42 Department of Internal Medicine V, Division of Hematology/Oncology, Heidelberg University Hospital, 69120 Heidelberg, Germany; stefan.schoenland@med.uni-heidelberg.de

**Keywords:** autologous stem cell transplantation in multiple myeloma, complications, multiple myeloma

## Abstract

Background: The main goal of this post hoc analysis of the Collaboration to Collect Autologous Transplant Outcomes in Lymphoma and Myeloma (CALM) study was to evaluate the rate of short- and long-term infectious and non-infectious complications occurring after ASCT in patients with multiple myeloma (MM). Methods: The analysis included all patients with MM from the CALM study who underwent ≥1 ASCT. The primary endpoint of the analysis was to determine the rate of infectious and non-infectious complications after ASCT and to compare them in three time periods: 0–100 days, 101 days–1 year, and >1 year after the first transplant. Results: The analysis included a total of 3552 patients followed up for a median of 56.7 months (range 0.4–108.1). Complication rates decreased with the time from ASCT with 24.85 cases per 100 patient-years from day 0 to 100 days after the transplant, and <2.31 cases per 100 patient-years from the 101st day. At 100 days after ASC T, 45.7% of patients had complications, with infectious events being twice as frequent as non-infectious complications. Bacterial infections (6.5 cases per 100 patient-years, 95% CI: 6.1–7.0) and gastrointestinal complications (4.7 cases per 100 patient-years, 95% CI: 4.3–5.1) were the most common early events. The pattern of complications changed with time from ASCT. The presence of complications after ASCT was not associated with overall survival. Conclusions: Our data provide a solid basis for comparing ASCT-related complications to those caused by emerging treatments in multiple myeloma, such as CAR T-cell therapy and other immunotherapies.

## 1. Introduction

Multiple myeloma (MM) morbidity and mortality are related to the disease itself and its complications, as well as treatment-related effects. Despite numerous new classes of effective drugs, autologous stem cell transplantation (ASCT) remains the standard of care in MM for younger patients without significant co-morbidities. Improvements in drug therapy, stem cell mobilization and supportive care has led to a continuous increase in the number of autologous transplants for the treatment of MM in a wide population of patients.

ASCT may increase the risk of complications due to compromised immunity and organ function. The objective of this study was to define the epidemiology, risk periods and clinical predictors of infectious and non-infectious complications in a large cohort of MM patients treated with ASCT. To overcome limitations of representativeness of observations from single centers, we studied the data reported to the European Society for Blood and Marrow Transplantation (EBMT) registry in the era of modern therapies.

## 2. Materials and Methods

The Collaboration to Collect Autologous Transplant Outcomes in Lymphoma and Myeloma (CALM) study (NCT01362972) was an analysis of data collected in the EBMT registry on a cohort of patients with multiple myeloma and lymphoma receiving autologous transplants of peripheral blood using cells mobilized with one of the following regimens: plerixafor plus granulocyte colony-stimulating factor (G-CSF), plerixafor plus G-CSF plus chemotherapy, G-CSF alone or G-CSF plus chemotherapy. We included all consecutive patients with MM from the CALM study who underwent ≥1 ASCT in this cohort between 2008 and 2012. The primary outcomes of the study were previously published [1,2]. Here, we report the analysis of the data collected in the CALM study. The analysis aimed to study the complication rate and mortality of ASCT in the treatment of MM.

The primary endpoint of the analysis was to determine the rate of infectious and non-infectious complications after ASCT. The rates were computed in three periods: 0–100 days, 101 days–1 year, and >1 year after the first transplant. Additionally, rates of complications were analyzed after the second ASCT. Analyzed infectious complications included bacterial, viral, fungal, and parasitic infections, and non-infectious complications were divided into gastrointestinal, pulmonary, neuropsychiatric, and hepatic complications. Rates of complications were compared according to patients’ characteristics to identify factors related to higher rates of complications.

Incidences of non-relapse mortality (NRM) and overall survival (OS) were studied to determine if complications are prognostic for long-term outcomes.

The study was performed in accordance with the principles of the Declaration of Helsinki and approved by the Transplant Complications Working Party of the EBMT, a non-profit scientific society representing more than 600 transplant centers mainly located in Europe. All patients whose transplant data are reported by participating centers provided informed consent for transplant-related data to be used for research purposes.

Data collection and outcome analysis followed EBMT registry-based studies and statistical guidelines [3]. Patients’ characteristics, types of complications, number of patients with recurrent complication and causes of death were summarized using counts and percentages. Rates of complications were defined as the number of events relative to the duration of the observation (rate of complications for 100 person-years). Rates of complications with 95% confidence intervals (CI) were provided in the three different periods: 1–100 days; 101 days–1 year; and >1 year after the first transplant and second transplant, respectively. Rates were calculated in each studied period, censoring for any death or second transplant within the time frame. The observation was censored at the time the patient received the transplantation of allogeneic stem cells. Rates were computed for any type of complications and separately for infections and non-infections. Comparisons between different patients’ characteristics were carried out using the rate ratio, which was computed as the ratio of the complication rates in the two groups. Multivariate analysis was performed to validate findings from univariate comparisons. Factors included in the multivariable analysis were gender (male vs. female), age at transplant (≥65 years vs. <65), renal function at transplant (impaired glomerular filtration rate ≤ 60 mL/min vs. normal glomerular filtration rate > 60 mL/min), status of disease at transplant (partial response or less vs complete response/very good partial response), Karnofsky Performance Score (KPS) at transplant (≥80 vs. <80). A *p*-value lower than 0.05 was considered statistically significant.

Overall survival was defined as the time from the date of ASCT to death from any cause. Patients alive at the time of the last follow-up were censored. OS curves were calculated using the Kaplan–Meier method and differences in groups of patients were assessed by the log-rank test. NRM was defined as the probability of dying without previous occurrence of a relapse, which was regarded as a competing event. NRM was estimated with a cumulative incidence function and compared between patients who had an infection and those who did not.

## 3. Results

### 3.1. Patient Characteristics

A total of 3762 patients from the CALM study fulfilled the general inclusion criteria. Of these, 210 patients were excluded because of missing or unreliable data on relapse (*n* = 133), at the second transplant (*n* = 60) or at infection occurrence (*n* = 17). The final analysis included 3552 patients treated in the EBMT centers over 5 years from 2008 to 2012. Patient and treatment characteristics are presented in Table 1. The median interval between diagnosis and the first transplant was 7.1 months (range: 1.1–378.2 months).

### 3.2. Transplant Complications

Rates of complications were analyzed in the three different periods: 1–100 days, 101 days–1 year, and >1 year after the first transplant. Overall, 1624 (45.7%) patients experienced at least one complication in the first 100 days after the ASCT (Table 2). In this period, patients experienced a total of 2898 complication events. Around half of the patients (54.6%) had just one complication. Among 1624 patients, at least one infectious complication occurred in 1298 (79.9%), and at least one non-infectious complication occurred in 753 (46.4%) within the first 100 days after ASCT. Both types of complications occurred in 427 patients (26.3%).

Early bacterial infections (6.5 cases per 100 patient-years, 95% CI: 6.1–7.0) and gastrointestinal complications (4.7 cases per 100 patient-years, 95% CI: 4.3–5.1) were the most common among early infectious and non-infectious complications, respectively (Table 2). Recurrent bacterial infections within the first 100 days after the transplant and ≥2 bacterial infections occurred in 21.7% of patients. Viral, fungal and unspecific/unclassifiable infections occurring more than twice during the first 100 days after transplant affected 15.4%, 11.2% and 18.6% of patients with any infection, respectively. In most patients (>95%), each type of non-infectious complication occurred only once in the studied period, except pulmonary complications, which affected 13.9% of patients more than twice in the first 100 days after the transplant.

**Figure 1:**
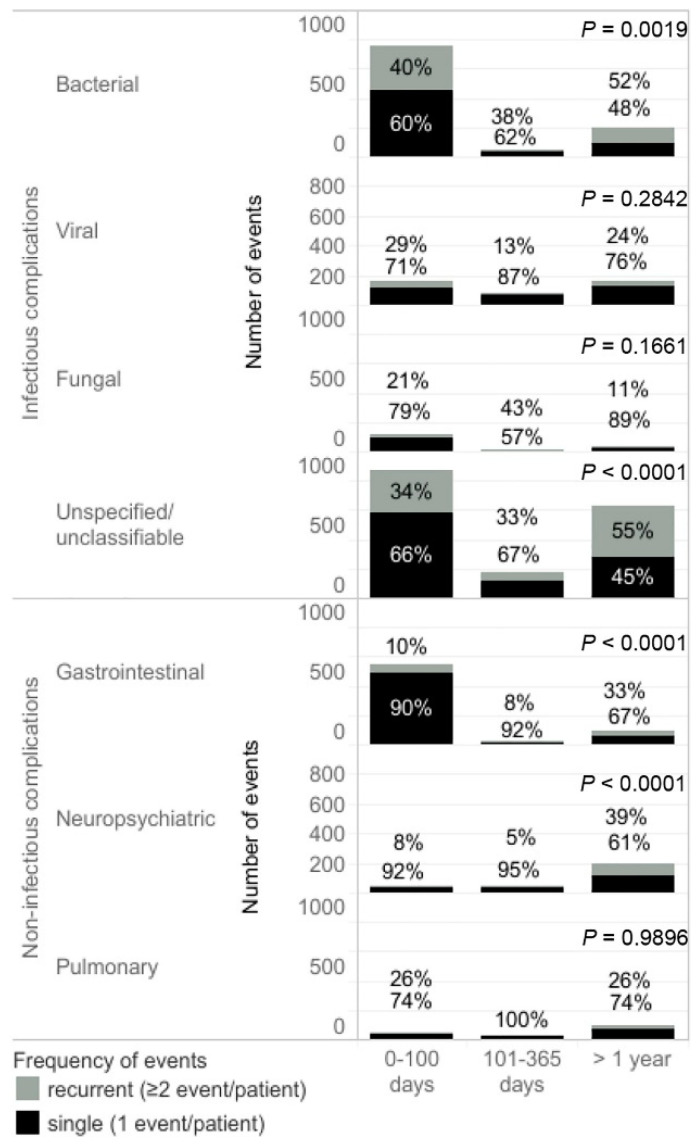
The number of complication events in periods of observation after the first transplant. Single complication indicates that the event occurred once in one patient. Recurrent complication indicates that the event occurred twice or more frequently in one patient during the observation period. *p*-values were calculated for proportion comparison between events occurring within 100 days immediately after the transplant and >1 year after the transplant.

The rate of complications decreased over time after the first transplant. Both infectious and non-infectious complications were significantly less common in the periods 101 days–1 year and >1 year after the transplant (Table 2). Viral infections became the most frequent infectious complications between the 101st day and 1 year after the transplant and had a similar frequency to bacterial infections one year after the transplant. Among non-infectious complications, neuropsychiatric events were most common 100 days after the transplant (Table 2). The percentage of recurrent bacterial infections was around 40% occurring right after the transplant and increased to 52% >1 year after the transplant (*p* = 0.0019). The proportion of recurrent unspecified infections >1 year after transplant was higher compared to the period early after transplantation (*p* < 0.0001) (Figure 1). Among patients with gastrointestinal and neuropsychiatric complications one year after the transplant, recurrent events occurred in 33% and 29% of cases, respectively, which was significantly more than the rates observed immediately after the transplant (*p* < 0.0001) (Figure 1).

For the subset of patients treated with the second transplant (*n* = 846), the rates of complications were analyzed. From the second transplant up to death/third transplant, ≥1 complication occurred in 392 cases. The overall complication rate was 4.4 per 100 person-years (95% CI: 4.2–4.7). The rate of infectious complications was 2.9 per 100 person-days (95% CI: 2.6–3.1) and 1.6 per 100 person-years (95% CI: 1.4–1.7) for non-infectious complications.

### 3.3. Complications by Patients’ Characteristics

Rates of complications occurring during the 100 days after the first transplant were compared according to patients’ characteristics (Table 3). The analysis underlines factors related to higher rates of complications. Multivariate analysis confirmed the main findings from univariate comparisons. Complications were more frequent in patients with KPS < 80, a disease stage lower than CR after induction treatment and impaired renal function. Male patients had a higher risk than female patients. Age was not significant in the multivariate analysis.

### 3.4. Survival

**Figure 2:**
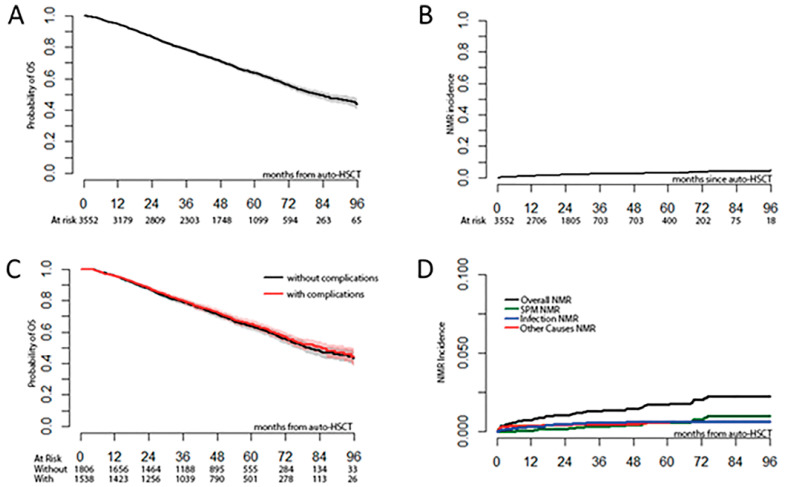
Survival and mortality outcomes after autologous stem cell transplantation. (**A**) Probability of overall survival. (**B**) Cumulative incidence of non-relapse mortality. (**C**) Overall survival of patients with and without complications. (**D**) Cumulative incidence of non-relapse mortality by its different causes.

After 56.7 months (range 0.4–108.1 months) median follow-up, the median OS in the total studied population was 82 months (95% CI: 77–89) (Figure 2A). At the time of observation, 1239 patients (34.9%) had died. A total of 210/1239 patients (16.9%) died due to causes other than disease progression or recurrence. In 181/1239 patients (14.6%), the specific cause of death was not reported. The main causes of death were disease relapse/progression (*n* = 883, 71.3%), infection (*n* = 83, 6.7%), secondary primary malignancy (*n* = 50, 4.0%), organ failure or toxicity (*n* = 33, 2.6%) and ASCT-related cause (0.7%).

The NRM incidences at 100 days, 1 year, and 5 years were 0.5%, 1.1% and 3.7%, respectively (Figure 2B). Infection-related NRM was most common; the 1 year incidence was 0.4 (95% CI: 0.2–0.6) vs 0.06 (95% CI: 0.0–0.1) for secondary malignancy and 0.3 (95% CI: 0.1–0.5) for other causes of NRM (Figure 2D).

There were no significant differences in OS among patients with and without complications. The median overall survival was 79.2 months (95% CI: 74.5–88.8) in patients without complications and 84.6 months (95% CI: 75.8–95.1) in patients with complications in the 100 days after the first transplant (*p* = 0.4646) (Figure 2C).

## 4. Discussion

The presented analysis is one of the largest single reports about ASCT complications in patients with MM. Some data were already available on ASCT complications during the time shortly after the transplant [4,5,6]. However, the short-term and long-term complication rates of ASCT have not been comprehensively assessed in a broad population of patients with MM. Here, we analyzed complications of ASCT in a large data set of patients treated in the CALM study with a long-term follow-up.

Transplant complications peaked in the early phase after the transplant with the highest incidence of bacterial infections and gastrointestinal complications. Each of these complications affected one-third of patients with any infectious and non-infectious complication, respectively. The high coincidence of bacterial infections and gastrointestinal complications seems to be associated with neutropenia and mucositis due to the cytotoxic effects of chemotherapy [4].

The overall incidence of infections within the first 100 days after the transplant was similar to that reported in earlier studies [5,6,7]. Developing complications up to 100 days after transplant did not affect long-term survival. The disease progression/relapse was the main cause of death after ASCT. Death because of organ failure was less common than in previously described cohorts [8].

The key to managing infection is understanding the specific risk factors and periods during which patients are at risk. Teh and coworkers indicated that bacterial infections in patients with MM have a bimodal distribution with peak incidence at a few months following diagnosis and a late peak 5–6 years later. Periods of a high incidence of infections were closely related to disease activity [9,10]. In the CALM study, the rate of both infectious and non-infectious complications substantially decreased 100 days after the transplant and remained low thereafter. Moreover, in the subgroup of patients receiving a second transplant, the rate of complications remained low after the second transplant compared to the first transplant. In the period between the 101st day and 1 year, viral infections were more frequent than bacterial infections. This pattern is similar to that observed in the general patient population, with the peak of viral infections being delayed compared to the highest incidence of bacterial infections [10]. One year after the transplant, recurrent bacterial infections were more frequent than immediately after the transplant, with a similar pattern observed among unspecified/unclassifiable infectious events. Typically, recurrent infections are associated with treatment nonsusceptibility, suggesting that resistance to antibiotics may play an important role in the incidence of long-term infectious complications.

Among non-infectious complications, after the first 100 days since ASCT, neuropsychiatric complications of ASCT became more frequent than initial gastrointestinal problems. These neuropsychiatric problems mostly include peripheral neuropathy, which can be secondary to the disease or its treatment.

In general, the factors increasing the risk of complications within 100 days after the transplant are similar to the factors compromising survival, as reported in previous studies, including low-performance status, disease stage at transplant and impaired renal function [11,12]. In addition, female patients are more at risk of complications than male patients. Information about a low NRM rate observed in the first 100 days after the ASCT is especially valuable for physicians discussing ASCT options with their patients.

The study has some limitations. The interpretation of results was limited due to the retrospective design of the study, and there was no available information on how heavily pretreated the patients were: maintenance therapy, immunoglobulin substitution, etc. Non-medical factors such as ethnic and socioeconomic status are known to influence long-term survival [13] but are not reported in the EBMT registry. This study did not explore the effect of the toxicity of different melphalan doses used in clinical practice due to its retrospective character. However, short- and long-term NRM rates were not significantly different between patients of the CALM study, who received conditioning with melphalan in doses of 200 or 140 mg/m^2^ [14]. Another important issue is that there are no data available regarding the severity of toxicity of events.

In summary, we found that complications frequently occur after ASCT for MM. Understanding their epidemiology and timing after ASCT is key to improving the management of patients and optimizing their quality of life. Our data provide a solid basis for comparing ASCT-related complications to those caused by emerging treatments in multiple myeloma, such as CAR T-cell therapy and other immunotherapies.

## Figures and Tables

**Table 1:** Patients’ demographics, disease and transplantation characteristics.

Characteristic	Patients, *n* (%)
No. of patients	3552
Male gender	2055 (57.9)
Age at the first transplant	
<65 years	2847 (80.2)
≥65 years	705 (19.8)
Diagnosis, subtype of MM	
IgA	684 (19.3)
Other Ig	1973 (55.5)
Light chain or NS	816 (23.0)
Missing	79
ISS stage at diagnosis	
I	836 (39.2)
II	776 (36.4)
III	519 (24.3)
Missing	1421
HCT-CI = 0 (low risk)	979 (63.4)
Missing	2007
KPS at the first transplant ≥80	3069 (94.8)
Missing	314
Renal function at the first transplant	
Normal	2585 (90.4)
Missing	693
Number of treatments before transplant	
1	1903 (64.8)
Missing	617
Disease status at the first transplant	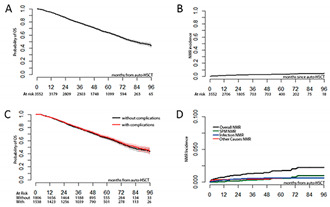
CR	557 (16.0)
Missing	62
Patients with second ASCT	846 (23.8)

CR, complete response; HTC-CI, Hematopoietic Cell Transplantation-Comorbidity Index; ISS; International Staging System; KPS, Karnofsky performance status; MM, multiple myeloma; NS, non-secretory.

**Table 2:** Types, rates and number of complications occurred after the first autologous stem cell transplantation.

Complication	0–100 Days after the Transplant	101 Days–1 Year after the Transplant	>1 Year after the Transplant
Patients with ≥1 complication, N	1624	376	846
Complications rate, cases per 100 patient-years (95% CI)			
Overall	24.85 (23.95–25.78)	2.31 (2.13–2.50)	2.22 (2.13–2.32)
Infectious complications	16.56 (15.82–17.32)	1.24 (1.11–1.38)	1.05 (0.99–1.12)
Non-infectious complications	8.29 (7.78–8.83)	1.07 (0.95–1.20)	1.17 (1.10–1.24)
Patients with ≥1 infectious complication, *n* (%)			
Bacterial	584 (36.0)	37 (9.8)	134 (15.8)
Viral	136 (8.4)	78 (20.7)	140 (16.5)
Fungal	107 (6.6)	11 (2.9)	34 (4.0)
Parasitic	2 (0.1)	0	1 (0.1)
Unspecified	710 (43.7)	141 (37.5)	424 (50.1)
Patients with ≥1 non-infectious complication, *n* (%)			
Gastrointestinal	517 (31.8)	24 (6.4)	72 (8.5)
Neuropsychiatric	49 (3.0)	42 (11.2)	148 (17.5)
Pulmonary	36 (2.2)	18 (4.8)	80 (9.5)
Hepatic	7 (0.4)	3 (0.8)	9 (1.1)

**Table 3:** Univariable and multivariable analysis of occurrence of complications occurring within first 100 days after the first transplant.

Characteristic	Complications Rate, Cases per 100 Patient-Years (95% CI)	Univariable Analysis		Multivariate Analysis	
		Rate Ratio (95% CI)	*p*-Value	Rate Ratio (95% CI)	*p*-Value
Gender					
Male	23.70 (22.55–24.90)	0.9 (0.83–0.97)	0.0042	0.88 (0.81–0.95)	0.003
Female	26.42 (25.00–27.90)				
Age					
<65 years	24.24 (23.25–25.27)	0.89 (0.81–0.97)	0.0095	0.98 (0.88–1.10)	0.754
≥65 years	27.32 (25.22–29.56)				
Diagnosis					
IgA	24.16 (22.16–26.29)	0.95 (0.86–1.05)	0.3075	-	-
Other Ig	25.45 (24.23–26.71)	0.97 (0.87–1.09)	0.6799		
LC or NS	24.79 (22.93–26.76)	1.03 (0.94–1.13)	0.5880		
HCT-CI					
Low	22.67 (21.05–24.38)	0.67 (0.6–0.75)	<0.001	-	-
Other	33.64 (31.04–36.39)				
KPS					
≥80	24.43 (23.47–25.42)	0.8 (0.68–0.94)	0.0075	0.70 (0.59–0.83)	<0.001
<80	30.54 (26.09–35.54)				
ISS					
I–II	26.43 (25.06–27.85)	0.93 (0.84–1.04)	0.191	-	-
III	28.37 (25.88–31.03)				
Renal function					
Normal	4.27 (23.23–25.35)	0.67 (0.59–0.75)	<0.001	0.68 (0.60–0.77)	<0.001
Impaired	36.32 (32.46–40.52)				
Treatments before transplant					
1	26.55 (25.29–27.87)	95 (0.88–1.03)	0.244	-	-
>1	27.88 (26.11–29.73)				
Disease status at transplant					
CR	17.87 (15.98–19.93)	0.68 (0.6–0.76)	<0.001	0.79 (0.69–0.91)	0.001
Other	26.45 (25.43 –27.50)				

CR, complete response; HTC-CI, Hematopoietic Cell Transplantation-Comorbidity Index; ISS, International Staging System; KPS, Karnofsky performance status; LC, light chain; MM, multiple myeloma; NS, non-secretory.

## Data Availability

Not applicable.
